# The biological roles of CD24 in ovarian cancer: old story, but new tales

**DOI:** 10.3389/fimmu.2023.1183285

**Published:** 2023-06-09

**Authors:** Yuanyuan Gu, Guannan Zhou, Xue Tang, Fang Shen, Jingxin Ding, Keqin Hua

**Affiliations:** ^1^ Department of Gynecology, The Obstetrics and Gynecology Hospital of Fudan University, Shanghai, China; ^2^ Department of Gynecology, Shanghai Key Laboratory of Female Reproductive Endocrine Related Diseases, Shanghai, China; ^3^ Changning Maternity and Infant Health Hospital, East China Normal University, Shanghai, China; ^4^ Department of Laboratory Medicine, Division of Biomolecular and Cellular Medicine, Karolinska Institutet, Stockholm, Sweden

**Keywords:** CD24, ovarian cancer, immunotherapy, Siglec 10, targeting therapy

## Abstract

CD24 is a glycosylphosphatidylinositol linked molecular which expressed in diverse malignant tumor cells, particular in ovarian carcinoma cells and ovarian carcinoma stem cells. The CD24 expression is associated with increased metastatic potential and poor prognosis of malignancies. CD24 on the surface of tumor cells could interact with Siglec-10 on the surface of immune cells, to mediate the immune escape of tumor cells. Nowadays, CD24 has been identified as a promising focus for targeting therapy of ovarian cancer. However, the roles of CD24 in tumorigenesis, metastasis, and immune escape are still not clearly demonstrated systematically. In this review, we i) summarized the existing studies on CD24 in diverse cancers including ovarian cancer, ii) illustrated the role of CD24-siglec10 signaling pathway in immune escape, iii) reviewed the existing immunotherapeutic strategies (targeting the CD24 to restore the phagocytic effect of Siglec-10 expressing immune cells) based on the above mechanisms and evaluated the priorities in the future research. These results might provide support for guiding the CD24 immunotherapy as the intervention upon solid tumors.

## Introduction

Ovarian cancer is acknowledged as one of the most lethal malignancies in reproductive system, which lacks of effective therapeutic strategies (1). The drug-resistance of ovarian cancer after surgery make it much more intractable (2). Increasing evidence suggested that immunotherapy could improve outcomes of patients in various cancers (3, 4). As a small heavy glycosylated antigen that overexpressed in ovarian carcinoma, CD24 expression is associated with the development, invasion, metastasis of cancers, as well as the immune evasion, which also gained increasing interest in the ovarian cancer therapy field (5). Here, we present an overview of CD24 in ovarian cancer as well as its potential applications in ovarian cancer therapy.

## CD24

It is widely reported that CD24 is a highly glycosylated protein linked to the plasma membrane via a glycosyl-phosphatidylinositol anchor (6). Traditionally, CD24 was acknowledged as a marker for pre-B lymphocytes (7). However, the expression of CD24 is different during the differentiation stages leading to late B cells (8, 9). Even though CD24 expression is also reported on various type of cells including myelocytes, keratinocytes, macrophages (10), renal tubular epithelial cells, muscle fibers, activated T cells, neurons, and ganglion cells. CD24 attracts the increasing interests for the expression in human cancer cells to mediate the cell invasion and immune evasion (11).

## CD24 in autoimmune diseases and other disorders

CD24 could act as an innate immune checkpoint in regulating host response to tissue injuries. It is well acknowledged that inactivating CD24 might confer to protection against autoimmune diseases such as systemic lupus erythematosus and multiple sclerosis. CD24 is expressed on a broad range of cells in the central nervous systems (CNS) and is related to the development of experimental autoimmune encephalomyelitis in mice. There are increasing studies focus on the association of a CD24 Ala/Val coding polymorphism with the susceptibility and progression of multiple sclerosis (12). It is reported that CD24 deficient mice could be more susceptible to danger associated molecular patterns but not pathogen-associated molecular patterns. CD24 could negatively regulate the stimulatory activity of high mobility group box 1, heat shock protein 70, as well as heat shock protein 90, and could inhibit the activation of nuclear factor kB (NF-kB) (13). In addition, Liu also reported that CD24-Siglec-E axis could play key role in obesity-related metabolic dysfunction. Silencing of the CD24-Siglec-E pathway could deteriorate the diet-induced metabolic disorders, including obesity, dyslipidemia, insulin resistance, and nonalcoholic steatohepatitis (NASH), which could be alleviated by CD24Fc treatment (14). While Siglec-G–CD24 axis was found that could regulate the severity of graft-versus-host disease in mice (15), it might be a promising strategy in mitigating GVHD. It is reported that CD24Fc fusion protein could amplify the Siglec-G signaling in donor T cells (since Siglec-G is a negative regulator of DAMP-mediated responses in innate immune cells (including T cells) *in vitro* and *in vivo*), and then ameliorated GVHD while preserving sufficient graft-versus-tumor (GVT) effects *in vivo (* 16). Recently, the anti-inflammatory effects of CD24 activation have also been explored as a strategy to counteract immune-related adverse events graft-versus-host disease, and sever inflammations, and recently in the COVID-19 treatment. A clinical study conducted by Tel-Aviv Sourasky Medical Center found that the exosomes-CD24 could release the symptom of severe COVID-19 infection (29 out of 30 patients treated with exosomes-CD24 fully recovered in five days) (17, 18). Thus, CD24Fc is also be accepted as an immunomodulator to reduce the exaggerated inflammatory response with tissue injuries. What’s more, it is reported that CD24Fc could protect simian immunodeficiency virus-infected Chinese rhesus monkeys against viral pneumonia (19). Some studies reported that CD24Fc could accelerate the clinical improvement of hospitalized patients with COVID-19 who are receiving oxygen support (20). In addition, CD24Fc could rapidly down-modulate the systemic inflammation and could restore immune homeostasis in SARS-CoV-2-infected individuals (21), suggesting that CD24 targeting inflammation in response to tissue injuries could provide a promising therapeutic option for patients hospitalized with COVID-19.

## CD24 in cancer immunotherapy

**Figure 1:**
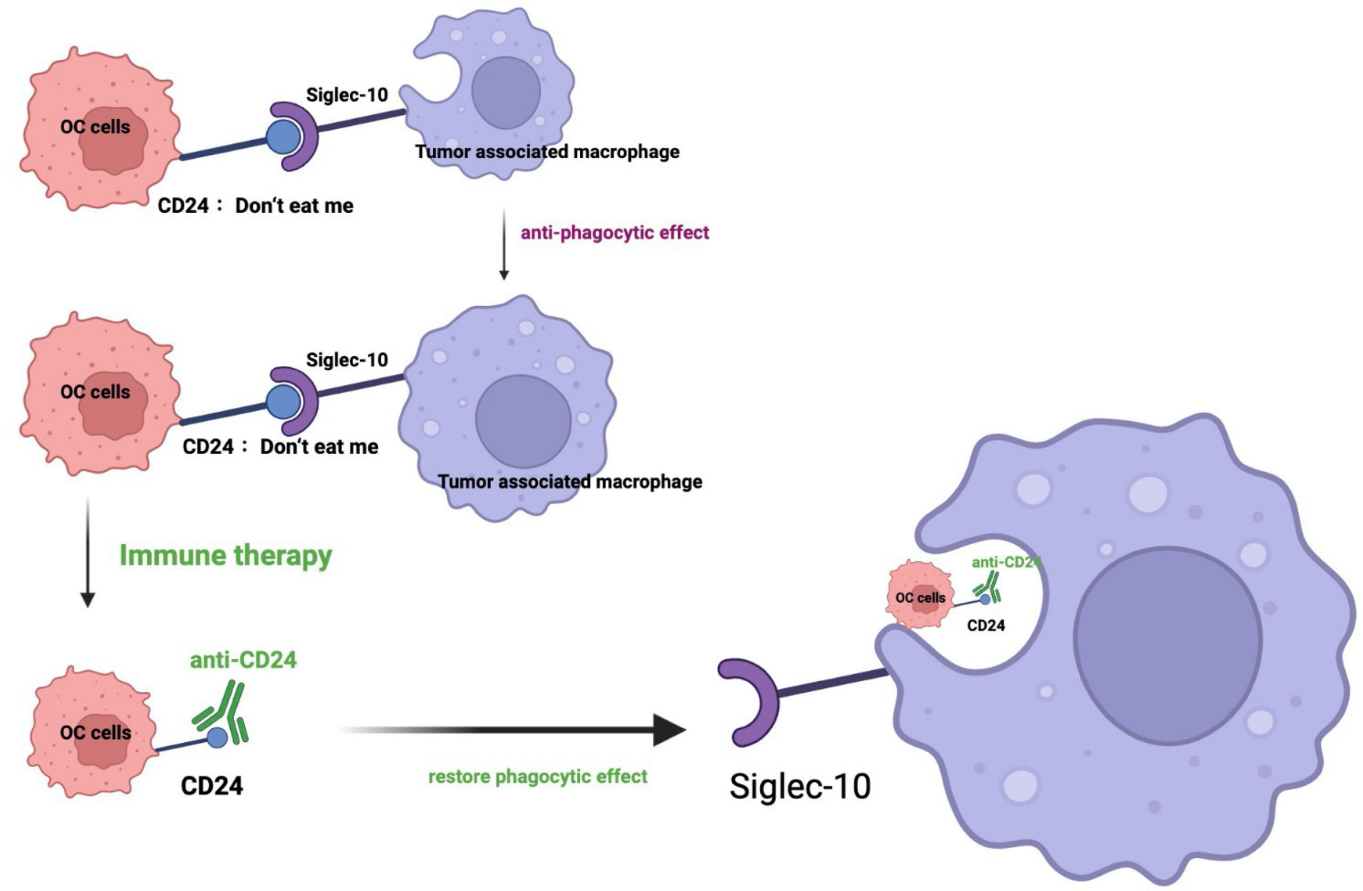
Schematic of CD24-Siglec-10 signaling in cancer immunotherapy. In this schematic image, the inhibitory receptor Siglec-10 recognizes and binds its ligand CD24 on ovarian cancer cells, resulting in the anti-phagocytic signaling cascades. Targeting the CD24 could restore the phagocytosis of immune cells. (OC: ovarian cancer; Siglec: sialic-acid-binding Ig-like lectin).

The immunotherapy has revolutionized cancer treatment in recent decades, some types of immunotherapies (including oncolytic virus therapies, cancer vaccines, cytokine therapies, adoptive cell transfer (ACT) as well as immune checkpoint inhibitors (ICIs)) have been recognized as promising therapies, and several of them (ACT and ICIs) have been applied in clinical practices (22). The CD24 expression in tumor cells is related to alterations in multiple oncogenic signaling pathways, including Src/STAT3, EGFR, WNT/β-catenin, and miRNA-related pathways. Based on the above theory, some CD24 targeting therapy studies have been conducted. Some malignancies have been reported that connect with Siglec-10 and employ the CD24/Siglec-10 interaction as a means of tumor immune evasion (23). The expression of CD24 in triple-negative breast cancer could help the cancer cells achieve the immune evasion through CD24-Siglec-10 signaling to inhibit the function of Tumor-associated macrophages (TAMs) (6) (**Figure 1**). The CD24–Siglec-10/G interaction constitutes an innate checkpoint that regulates inflammation triggered by danger-associated molecular patterns (DAMPs) (24) and cancer pathogenesis. The blockade of CD24 could suppress the oral squamous cell carcinoma (OSCC) growth while reducing the TAMs number and enhancing the T cell number *in vivo (* 25). The G7 monoclonal antibody designed against CD24 has demonstrated some antitumor activity (26). Recently, Lai reported that NPM/B23 could induce the expression level of CD24 in endometrial cancer cells to help cancer cells escape from the phagocytosis effect from macrophages. On the contrary, silencing the NPM/B23 could enhance the phagocytic effect by macrophages via reducing the expression level of CD24 on the cell surface (27). Above all, it is widely accepted that CD24 is playing vital role in cancer immunotherapy. In addition, it is reported that CD24Fc could ameliorate the immune-related adverse events while preserving anti-tumor therapeutic effect in human CTLA-4 knock-in (*Ctla4^h/h^*) and humanized NSG mouse models (28).

## The expression of CD24 in ovarian cancer

It is well demonstrated that the expression of CD24 is related to the development of ovarian cancer in many studies as below (**Table 1**). In the recent decades, immunotherapy with the aim to stimulate the endogenous immune response against ovarian cancer cells has been a new frontier of the anticancer treatment (39). For example, the blockade of the programmed death 1 (PD1) pathway, has been applied at different steps of clinical practices against cancers (40) [including ovarian cancer (41)]. There are convincing data supporting that the immunotherapy of CD24 is of significance in the gynecological malignancies. Welsh et at reported the CD24 expression in the ovarian cancer specimens in 2001 (42). As the study focus more on the relationship between CD24 and ovarian cancer, there are increasing evidence to illuminate the vital role of CD24 in the development of ovarian cancer. Kristiansen reported that the expression of CD24 in cytoplasm is an independent molecular marker for reduced patient survival in ovarian cancer (29). Peter Altevogt found that Ovarian carcinoma ascites derived exosomes contain CD24, which might enhance the tumor invasion ability into the stroma (31). Moulla reported that high grade ovarian carcinomas could express higher level of CD24, when compare with low grade ovarian carcinomas. Similarly, other studies also reported that in the CD24 level in metastatic ovarian carcinomas are higher than ovarian carcinomas without metastases (32). Gao and Wei observed the CD24 expression is relevant to the stemness of ovarian stem cancer cells (34, 35). It is reported that integrase-derived peptides together with CD24-targeted lentiviral particles inhibit the growth of CD24 expressing cancer cells, which suggests that the CD24 could be an effective targeting tools for drug delivery (43). Additionally, surgeons reported that CD24-targeted intraoperative fluorescence image could be a promising application in improving debulking surgery of ovarian cancer. This result depicted that CD24 based fluorescence image-guided surgery (FIGS) could improve the tumor detection and resection, and subsequently reduce the post-operative ovarian carcinoma tumor burden (44).

**Table 1:** The expression of CD24 in Ovarian Cancer.

Researchers	Years	Fields	Conclusions	Ref
Kristiansen et al.	2002	CD24 expression in OC	CD24 expressed in invasive ovarian carcinomas but no expression on surface epithelium of normal ovaries	(29)
Geunghwan Ahn et al.	2005	CD24 expression in advanced ovarian serous borderline tumors	the increase of CD24 is an independent factor in ovarian serous adenocarcinomas for shortened survival rate	(30)
Peter Altevogt et al.	2007	CD24 expression in ascites	Ovarian carcinoma ascites derived exosomes contain CD24	(31)
Moulla et al.	2013	CD24 expression in different pathological subtype of OC	Increased CD24 expression in borderline and malignant ovarian carcinomas	(32)
Bretz et al.	2012	CD24 over-expression or depletion in OC cell lines	CD24 could affect STAT3 phosphorylation via activation of Src kinase	(33)
Gao et al.	2010	CD24 in ovarian Cancer Stem Cells	CD24 is relevant to the stemness in OC	(34)
Wei et al.	2010	CD24 in ovarian Cancer Stem Cells	The CD24+ CSCs exhibit EMT phenotype	(35)
Barkal et al.	2019	CD24 in ovarian cancer	CD24 can be the dominant innate immune checkpoint in ovarian cancer	(36)
Nagare et al.	2020	CD24 in ovarian Cancer Stem Cells	CD24 may be a putative Cancer Stem Cells marker in ovarian cancer	(37)
Nagy et al.	2019	CD24 in ovarian cancer tissue samples	CD24 expression is association with FIGO classification	(38)

## Immunotherapy of CD24 in ovarian cancer treatment

It is widely accepted that cancer cells could avoid the clearance from macrophages through the overexpression of “don’t eat me” signals. These “don’t eat me” signals are anti-phagocytic surface proteins, including CD47 (45–47), programmed cell death ligand 1 (PD-L1) (48) as well as CD24. Briefly, CD24/Siglec-10 interaction could play vital role in tumor immune evasion by inhibiting macrophage mediated phagocytosis (36) as well as natural killer (NK) cell cytotoxicity.

## CD24-Siglec-10 axis and immune evasion

Although the TCGA data showed that almost all the tumors expressed the high level of CD24, the ovarian cancer showed the largest upregulation (36). Mostly, phagocytic signals are expressed on the tumor surface, including tumor-associated antigen, endoplasmic reticulum chaperone, calreticulin (49) and so on. Recently, several studies reported that some anti-phagocytic signals also exist on the surface of tumor cells, including CD47 (45), PD-L1 (50), and CD24. Ovarian cancer cells expressed CD24 could achieve the immune evasion through interacting with the receptor called sialic-acid-binding Ig-like lectin 10 (Siglec-10 has been reported to interact with the highly sialylated form of CD24), which is expressed by tumor associated macrophages (TAM) (**Figure 1**). CD24 could protects ovarian cancer cells from macrophage attack *in vitro*, and *in vivo (* 36). Mechanically, the signaling axis of CD24–Siglec-10 interactions could regulate macrophage-mediated antitumor immune responses. This anti-phagocytosis signal is related to macrophage signaling based on immunoreceptor-tyrosine-based inhibition motifs and essentially avoid the surveillance and clearance of macrophages. In a co-culture model, stable genetic ablation of CD24 could increase the phagocytosis of cancer cells by Siglec-10 positive M2-like macrophages. In addition, SIGLEC10 knockout could enhance the phagocytic ability of macrophages towards cancer cells. These results verified that the CD24–Siglec-10 interaction plays a role of anti-phagocytic effect (**Figure 1**). However, CD24 could bind to Siglec-10 specifically, but not to Siglec-3 and Siglec-5 (51). Another study reported that a novel anti-CD24 chimeric antigen receptor (CAR) as an immunotherapeutic approach against ovarian cancer cells and cancer stem cells. The codon-optimized third-generation CAR containing the highly active single chain variable fragment (scFv) “SWA11” against CD24 could engineer the NK cells with high cytotoxic activity against CD24-positive ovarian cancer cell lines (52). Also, CD24 blockade has shown promising results in preclinical studies (**Table 2**). Also, it is reported that liposomal cisplatin with a red fluorescent substance cyanine 5.5 and mortified with anti-CD24 monoclonal antibody, called (CD24-GL-CDDP-Cy5.5) could improve of the delivery of cisplatin (55) via increasing the targeting the CD24 positive ovarian cancer cells (55). CD24 is becoming a compelling therapy target for the immunotherapy of cancer due to its important role in both the regulation of the immune response as well as the tumorigenesis process (56). Increasing trials is focusing on the strategy not only on blocking the CD24 on the surface of tumor cells, but also on targeting the CD24 and Siglec-10 genes, as well as targeting on the binding of CD24-Siglec-10 signal pathway (new insight and strategy in tumor immunity therapy field).

**Table 2:** CD24 relevant preclinical studies.

Production	Briefly functions	Briefly mechanism	Ref
SW11	Inhibiting the tumor growth	Inhibition of Src/STAT3 signaling	(53)
SW11	Inhibiting the tumor growth	Regulation of intra-tumoral cytokine microenvironment	(54)

## CD24 and cancer metastatic and progression

Since CD24 is highly expressed in various tumor cells, it is related to the invasion and progression of tumor cells. The CD24-expressing tumor cells could identify and combine the P-selectin on platelets, leading to tumor cells excrete and subsequently metastasis (57). In addition, CD24 could activate the signal factors in the lipid rafts microdomains (58), such as Src kinase. The activated Src kinase could lead to the tumorigenesis. CD24 could inhibit the tissue factor pathway inhibitor-2 (TFPI-2) by regulating the Src related pathway, and then promote the metastasis of tumor cells (33). Some studies showed that the CD24 expression is related to the hypoxia condition in tumor microenvironment. HIF acts as a transcription factor to induce CD24 expression at the transcriptional level (59), and promote the cancer invasion (60). Also, signals derived from non-coding RNAs (ncRNAs) could regulate the expression of CD24. The lncRNA-H19 could reduce the expression of cell-surface CD24 in tumor cells and regulate the tumor immune escape (61). Also, microRNA-34a could reduce the expression of CD24 and Src at the post-transcriptional level, and then regulate the tumor immune escape (62). In addition, Wnt/β-catenin pathway could regulate the expression of CD24 in cancer cells. The β-catenin could inhibit the tumor immune escape by down-regulating the expression of CD24 (63). Thus, CD24 is becoming a therapeutic target. SWA11 monoclonal antibody could bind CD24-expressing cells specifically and then inhibit the proliferation of tumor cells (64). Some study also reported that with the help of anti-CD24 antibody, the chemotherapy could exert more advantage when targeting chemotherapy-resistant tumor stem cells (54).

## Summary and perspectives

Ovarian cancer is the most lethal gynecological carcinoma due to the difficulty of early diagnosis and further drug resistance. Recent years, increasing targeting immune therapies have been applied due to its specificity towards cancer cells. Even though several molecular markers have been known for ovarian cancer targeting therapy, there are still some limitations for widely applications. Exploring novel molecular targets is urgent in cancer targeting therapy. CD24 could act as anti-phagocytic signal (“don’t eat me” signaling protecting cancer cells away from phagocyting effect by Siglec-10-expressing macrophages) in ovarian cancer cells and could act as a blockade molecular in the cancer immunotherapy. In conclusion, CD24 is promising surface marker and potential targeting molecular in ovarian cancer.

## Author contributions

YG: Writing-Original Draft. GZ: Writing-Original Draft and Editing. XT: Writing-Original Draft and Editing. FS: Writing-Review. JD: Review and Editing. KH: Writing-Review and Editing, Supervision. All authors contributed to the article and approved the submitted version.
